# The association between device-measured sitting time and cardiometabolic health risk factors in children

**DOI:** 10.1186/s12889-024-18495-w

**Published:** 2024-04-12

**Authors:** Ana María Contardo Ayala, Nicola D. Ridgers, Anna Timperio, Lauren Arundell, David W. Dunstan, Kylie D. Hesketh, Robin M. Daly, Jo Salmon

**Affiliations:** 1 Institute for Physical Activity and Nutrition (IPAN), School of Exercise and Nutrition Sciences, Deakin University, 221 Burwood Highway, Geelong, Victoria Australia; 2 Alliance for Research in Exercise, Nutrition and Activity (ARENA), Allied Health and Human Performance, University of South Australia, Adelaide, South Australia Australia

**Keywords:** Children, Health outcomes, Physical activity, Sedentary behavior

## Abstract

**Background:**

There is limited evidence of the associations between postural-derived sitting time, waist-worn derived sedentary time and children’s health and the moderation effect of physical activity (PA). This study examined associations of children’s device-measured sitting time with cardiometabolic health risk factors, including moderation by physical activity.

**Methods:**

Cross-sectional baseline data from children (mean-age 8.2 ± 0.5 years) in Melbourne, Australia (2010) participating in the TransformUs program were used. Children simultaneously wore an *activ*PAL to assess sitting time and an ActiGraph GT3X to assess sedentary time and physical activity intensity. Cardiometabolic health risk factors included: adiposity (body mass index [BMI], waist circumference [WC]), systolic and diastolic blood pressure (SBP, DBP), high-density lipoprotein (HDL), low-density lipoprotein (LDL), cholesterol, triglycerides, fasting plasma glucose (FPG), serum insulin, and 25-hydroxyvitaminD (25[OH]D). Linear regression models (*n* = 71–113) assessed associations between sitting time with each health risk factor, adjusted for different PA intensities (i.e. light [LIPA], moderate-vigorous intensities [MVPA], separately on each model), age, sex, adiposity, and clustering by school. Interaction terms examined moderation. The analyses were repeated using device-measured sedentary time (i.e. ActiGraph GT3X) for comparison.

**Results:**

Sitting time was positively associated with SBP (*b* = 0.015; 95%CI: 0.004, 0.026), DBP (*b* = 0.012; 95%CI:0.004, 0.020), and FPG (*b* = 0.001; 95%CI: 0.000, 0.000), after adjusting for higher PA intensities. The association between sitting time and insulin (*b* = 0.003; 95%CI: 0.000, 0.006) was attenuated after adjusting for higher PA intensities. When the models were adjusted for LIPA and MVPA, there was a negative association with LDL (*b*=-0.001; 95%CI: -0.002, -0.000 and *b*=-0.001; 95%CI: -0.003, -0.000, respectively). There was a negative association of sedentary time with WCz (*b*=-0.003; 95%CI: -0.005, 0.000) and BMIz (*b*=-0.003; 95%CI: -0.006, -0.000) when the models were adjusted by MVPA. Sedentary time was positively associated with triglycerides (*b* = 0.001; 95%CI: 0.000, 0.001) but attenuated after adjusting for MVPA. No evidence of moderation effects was found.

**Conclusions:**

Higher volumes of sitting and sedentary time were associated with some adverse associations on some cardiometabolic health risk factors in children. These associations were more evident when sitting time was the predictor. This suggests that reducing time spent sitting may benefit some cardiometabolic health outcomes, but future experimental research is needed to confirm causal relationships and identify the biological mechanisms that might be involved.

**Trial registration:**

Australian New Zealand Clinical Trials Registry: ACTRN12609000715279.

**Supplementary Information:**

The online version contains supplementary material available at 10.1186/s12889-024-18495-w.

## Background

Sedentary behaviours, characterised as waking behaviours with an energy expenditure ≤ 1.5 metabolic equivalents (METs) while in a sitting or reclining posture [1], have been associated with unhealthy cardiometabolic profiles in adults [2]. There has been significant interest in examining both cross-sectional and longitudinal associations between sedentary time volumes and patterns typically assessed using hip-worn devices, with a range of children’s health outcomes including adiposity markers [3–5], cardiorespiratory fitness [3], cardiometabolic risk [6] among children and adolescents. However, these associations have been mixed [4, 7–12]. Some studies amongst youth have reported positive associations between sedentary time and cardiometabolic risk factors, which attenuate when adjusted for moderate- to vigorous-intensity physical activity (MVPA) [13, 14]. In contrast, some studies have shown associations remain unchanged after adjustment for MVPA [15, 16]. This may partly relate to the assessment used, which traditionally relies on accelerometer cut-points to estimate periods of ‘little too low movement’ and does not differentiate between postures when sitting or standing still [17, 18].

While a novel computational method has been developed that classifies sitting posture from hip-mounted accelerometers among children [19], postural-detection devices (e.g. *activ*PAL) can differentiate between sitting and standing but have rarely been used to explore associations between sitting time and cardiometabolic risk factors among youth [20, 21]. In the studies that have assessed sitting using postural devices, Contardo Ayala et al. [21] found no cross-sectional associations between *activ*PAL-measured sitting time and adiposity markers (body mass index [BMI] or waist circumference [WC] z-scores) among 219 adolescents (age = 14.9 ± 1.6 years). While some evidence of associations has been observed for components of sitting patterns (i.e., breaks, prolonged sitting) with adiposity and high-density lipoprotein cholesterol (HDL) among 118 children (aged 11–12 years) [20], total sitting volume was not reported due to collinearity in the model. Further research using postural devices is needed to better understand associations between sitting time and cardiometabolic risk factors in children.

Different analytical methods have been used to examine associations between movement behaviours and cardiometabolic health [14]. Historically, the focus has been on MVPA and sedentary time, often with adjustments for other intensities and assessed using one device [12]. However, little research has examined the potential health benefit of light-intensity physical activity (LIPA), and the use of different devices to capture sitting and physical activity of different intensities on these associations. It is important to understand the potential health benefits of LIPA, as it represents a substantial portion of daily physical activity in adolescents [22]. In addition, examining whether physical activity moderates associations between sitting and health, and which intensity of activity moderates the associations to a greater extent, has rarely been explored despite this potentially providing insights into how much physical activity is needed to counteract the detrimental effects of too much sitting. However, to our knowledge, the moderation effect of different physical activity intensities on the association between sitting time and health markers has only been reported in one study [21]. That study found that LIPA and not MVPA moderated the relationship between sitting and adiposity markers (i.e. BMI and WC) in adolescents [21], with negative associations between sitting and adiposity markers in those who engaged in greater amounts of LIPA. These findings suggest that increasing time spent in LIPA may attenuate the potential deleterious association of high levels of sitting on health. However, whether different PA intensities moderate relationships between sitting and other cardiometabolic risk factors among children has yet to be examined in this way.

The aim of this study was to examine the cross-sectional associations between children’s device-measured sitting time (i.e. *activ*PAL3) and cardiometabolic risk factors and to determine whether physical activity of varying intensities moderated associations between sitting time and cardiometabolic risk factors. We also repeated the analyses using device-measured sedentary time (i.e. ActiGraph GT3X) for comparison.

## Methods

### Study population

Baseline data from the TransformUs randomised controlled trial [23] were analysed. TransformUs was an 18-month, four-arm cluster-randomized controlled trial within primary schools in Melbourne, Australia (2010-12), aiming to increase children’s physical activity, decrease sedentary behaviour, and optimize health outcomes [23, 24]. TransformUs was approved by the Deakin University Human Research Ethics Committee Group (141–2009), Victorian Department of Education and Early Childhood Development (2009_000344) and Catholic Education Office (1545).

Recruitment details have been published previously [24]. In brief, schools within a 50 km radius from the Melbourne Central Business District were identified. The school selection criteria included: enrolment greater than 300 students, having at least two grade three classes, being co-educational (i.e. no single-sex schools) and being located in the first, third or fifth quintile of the Socio-economic Index for Areas (SEIFA; for socio-economic diversity) [25]. In total, 20 schools were recruited. All grade three students were invited to consent to the opt-in evaluation assessments (*n* = 1606). In total, 599 parents provided written informed consent for their child to participate in evaluation assessments (37%), and of these, 532 consented to wear an *activ*PAL. Due to equipment availability, a random sample of 216 children wore an *activ*PAL at baseline. Parental consent to collect children’s blood pressure and blood samples was obtained for 477 and 219 children, respectively. Baseline data collection occurred between February to June 2010.

### Measures

#### Demographics

Children self-reported their sex and age in a survey completed during class time.

#### Physical activity, sitting and sedentary time

Participants were asked to concurrently wear two devices during waking hours (except for water-based activities) for eight days. Children wore an *activ*PAL3 inclinometer (PAL Technologies Ltd, Glasgow, UK) on the front of their right thigh (at the mid-point), attached via an elastic belt. The *activ*PAL3 is considered a valid measure of sitting [26]. Children also wore an ActiGraph GT3X (ActiGraph LLC, Pensacola, FL., USA) on an elastic belt on their right hip. The ActiGraph is commonly used to quantify physical activity in children [27]. All data were extracted using manufacturer software (*activ*PAL Professional v7.2.29 and Actilife v6.11.18) and processed using a customised Microsoft Excel macro in 15-second periods. Consecutive zero counts for 20 min were used as non-wear time for both devices [28, 29]. For each device, data were included in the analysis if participants had at least four valid days (including a weekend day), which was defined as ≥ 8 h/day of wear time for weekdays and ≥ 7 h/day for weekend days [30, 31].

From the *activ*PAL data, the total volume of sitting was computed as the average duration per valid day (mins/day). From the ActiGraph data, Freedson age-adjusted cut-points [32] were used to obtain average time spent in sedentary time (< 100 counts/minutes), LIPA (i.e. 101 counts/minute to 3.99 METs) and MVPA (≥ 4 METS for those < 18years, based on age-specific thresholds [e.g. for a 8 year old is 802 counts/minute]) per valid day. LIPA was further dichotomised into low light-intensity physical activity (low-LIPA) (i.e. 101–799 counts/minute) and high light-intensity physical activity (high-LIPA) (i.e. 800 counts/minute to 3.99 METs). The cut-point of > 800 counts/min was based on previous research as it captures static LIPA, such as standing as high-LIPA [17, 33]. Prior to the analysis, all *activ*PAL and ActiGraph-derived outcome variables were standardised (to account for variations in the amount of time individuals wear the device) according to total wear time during the period of interest as follows: (duration of ‘X’ within waking hours/wear time within waking hours) multiplied by 960 min, where ‘X’ is the activity (e.g., sitting, LIPA, low-LIPA, high-LIPA and MVPA) and waking hours are equivalent to 960 min–16 h.

#### Anthropometric measures

Measurements were taken at the schools by trained researchers using standardised protocols. Stature was measured to the nearest 0.1 cm using portable stadiometers (SECA 0123, SECA, Hamburg, Germany) and body mass to the nearest 0.1 kg using portable electronic scales (InnerScan 50; TANITA, Illinois, USA). WC was assessed using a flexible measuring tape at the narrowest, or middle, point between the bottom rib and the iliac crest. Two measurements were taken, with a third measure taken when a difference of over 0.5 cm (stature), 0.5 kg (body mass) and 1 cm (WC) was noted. BMI (kg/m^2^) was calculated from body mass and stature, and participants were categorised according to the International Obesity Task Force definitions of healthy weight or overweight/obese [34]. BMI z-scores (BMIz) were calculated and converted to z-values, standardized for age and sex, using the World Health Organization Child Growth Standards [35]. WC z-scores (WCz) were calculated from raw anthropometric data using Stata function with UK growth chart (zanthro) [36]. Australian percentile curves for WC were utilised to determine age- and sex-specific waist circumference percentiles, where a WC ≥ 90th percentile was used to classify obesity and overweight was defined as a WC ≥ 75th percentile but less than the 90th percentile [37].

#### Blood pressure

Participants were asked to sit quietly for two minutes, then blood pressure (BP) was measured from the right arm using a paediatric cuff (OMRON HEM-907, Kyoto, Japan). Three measures were taken one-minute apart, on two different occasions one week apart. Average systolic and diastolic BP were calculated from four measures after removing the first measurement from weeks one and two [38].

#### Blood biomarkers

Following an overnight fast, a morning blood sample was collected from each child with parental consent by a trained phlebotomist at a local commercial pathology clinic (the same pathology company was used for all blood samples). All children were provided with a dermal anaesthetic topical cream (EMLA®) to apply 1 h before the blood collection. The following biomarkers were assessed by the pathology laboratory using standard laboratory techniques: HDL-cholesterol, LDL- cholesterol, total cholesterol, triglycerides, fasting plasma glucose (FPG), serum insulin, and serum 25-hydroxyvitamin D (25[OH]D). All assays were performed according to manufacturer’s instructions, and all samples were run in duplicate. Biomarker ranges considered as acceptable are presented in the Additional file 1.

### Statistical analysis

Statistical analyses were conducted using Stata 18.0 (Stata-Corp LPD46XX). Statistical significance was set at *p* < 0.05. Shapiro–Wilk test were used to check the normality of the data. Pearson’s correlations were used to test for multicollinearity, confirming the absence of collinearity between the variables studied (i.e. the correlation between sitting [from *activ*PAL] and each physical activity intensity variable [from ActiGraph] ranged between − 0.06 and 0.05). Collinearity was confirmed between sedentary time [from ActiGraph] and LIPA [from ActiGraph].

The main association of *sitting *(minutes/day) with cardiometabolic risk factors was examined using separate regression models (Model A) for each cardiometabolic health marker (11 models in total). The associations with cardiometabolic risk factors were further explored by adjusting for each PA intensity (LIPA and MVPA) separately, age, sex, adiposity (BMIz), season in which the assessment was conducted, and clustering by school (Models B to E). Moderation by LIPA, low-LIPA, high-LIPA, and MVPA were also examined by adding interaction terms to the respective main regression models and checking *p* values. The main association of *sedentary time (minutes/day)* with cardiometabolic risk factors was examined using separate regression models (Model A) for each cardiometabolic health marker (11 models in total). The associations with cardiometabolic risk factors were further explored by adjusting for MVPA, age, sex, adiposity (BMIz), season in which the assessment was conducted, and clustering by school (Model B). Moderation by MVPA was also examined. All moderator variables were centered around the individual variable mean.

## Results

*activ*PAL data: In total, 113 children provided valid *activ*PAL data, of whom 103 had valid ActiGraph data. For the analyses, 113 children had anthropometric measures, 111 had BP measures, and 71 had blood biomarkers. For the analysis of the ActiGraph data, 334-335 children had anthropometric measures, 323 had BP measures, and 159 had blood biomarkers.

Children (mean ± SD, age 8.1 ± 0.4 years, 58% girls) spent an average of 59% of their waking hours sitting, 41% in LIPA (33% and 8% in low and high LIPA, respectively), and 4% in MVPA (Table 1). All participants had their biomarkers in the acceptable range.

**Table 1:** Demographic characteristics and cardiometabolic risk factors of the participants included in the analysis

		*activ*PAL sample		ActiGraph sample
	N	Mean (SD)	N	Mean (SD)
Age (years)	113	8.1 (0.4)	338	8.2 (0.5)
Females, n (%)	113	58 (50.9)	338	197 (58.3)
BMI (kg/m^2^)	113	17.3 (2.4)	334	17.5 (2.9)
BMIz	113	0.7 (1.0)	334	0.7 (1.2)
*BMI categories (%)*	113		334	
Healthy weight		71.8		73.9
Overweight/obese		28.2		26.1
WC (cm)	113	59.2 (5.9)	335	59.8 (6.9)
WCz	113	0.8 (1)		
*WC categories (%)*	133		335	
Healthy weight		63.7		65.9
Overweight/obese		36.3		34.0
SBP (mmHg)	111	102.2 (9.5)	327	102 (9.1)
DBP (mmHg)	111	59.8 (8.5)	327	60.6 (8.4)
Cholesterol (mmol/L)	71	4.6 (0.7)	160	4.6 (0.8)
HDL (mmol/L)	71	1.7 (0.3)	160	1.7 (0.3)
LDL (mmol/L)	71	2.5 (0.7)	160	2.6 (0.7)
Triglycerides (mmol/L)	71	0.7 (0.2)	160	0.7 (0.3)
FPG (mmol/L)	71	4.5 (0.3)	160	4.6 (0.5)
Insulin (uU/ml)	71	5.4 (2.9)	160	6.0 (3.8)
Vitamin D (nmol/L)	71	76.7 (23.7)	160	72.6 (25.7)
Sitting time (min/day) ^(⁎)^	103	567.4 (108.6)	103	556.1 (111.9)
Sedentary time (min/day) ^(⁑)^	103	529.8 (55.7)	340	535 (51.8)
Total-LIPA (min/day) ^(⁑)^	103	391.0 (48.5)	340	388.7 (46.7)
Low-LIPA (min/day) ^(⁑)^	103	314.2 (39.2)	340	313.2 (38.9)
High-LIPA (min/day) ^(⁑)^	103	77.7 (18.1)	340	75.6 (16.8)
MVPA (min/day) ^(⁑)^	103	38.2 (18.7)	340	36.2 (17)

*Abbreviations* BMIz = body mass index z-score; WCz = waist circumference z-score; SBP = Systolic Blood Pressure; DBP = Diastolic Blood Pressure; HDL = High-Density Lipoprotein; LDL = Low-Density Lipoprotein; FPG = fasting plasma glucose; Vitamin D = 25hydroxyvitaminD; CRP = C-reactive protein; total-LIPA = Light-Intensity Physical Activity; low-LIPA = Low Light-Intensity Physical Activity (101 − 3.99 METS); low-LIPA = Low Light-Intensity Physical Activity (101–799 counts per minute); high-LIPA = High Light-Intensity Physical Activity (800 counts per minute– 3.99 METS); MVPA = Moderate- to- Vigorous-intensity Physical Activity (≥ 4 METS). Biomarkers references values: Cholesterol = 2.4–4.5 mmol/L; Triglycerides = 0.4–1.5 mmol/L; HDL = > 1.20 mmol/L; LDL = < 3.5 mmol/L; FPG = 3.6-6.0 mmol/L; Insulin = 0–17 uU/mL; 25-hydroxyvitamin D = 75–250 nmol/L

^(⁎)^ Data derived from *activ*PAL inclinometers

(⁑) Data derived from ActiGraph accelerometers

### Associations of sitting time with health risk biomarkers

Table 2 reveals a positive association between sitting time and SBP, DBP, and FPG, persisting even after adjustments for age, sex, adiposity, season in which the assessment was conducted, and various physical activity intensity variables in the model. Similarly, sitting time was positively associated with insulin, but this association was attenuated after adjusting for the physical activity intensity variables. There was a negative association with LDL in the LIPA (i.e. total LIPA and low-LIPA) and MVPA adjusted models.

**Table 2:** Associations between sitting time and cardiometabolic risk factors prior to and after adjustment for physical activity intensity

Dependent variable:		Model A	*p*		Model B: LIPA	Model C: Low LIPA	Model D: High LIPA	Model E: MVPA
	n	β (95% CI)	value	n	β (95% CI)	β (95% CI)	β (95% CI)	β (95% CI)
WCz	113	-0.000 (-0.002, 0.001)	0.862	103	0.000 (-0.001, 0.002)	0.000 (-0.001, 0.002)	-0.000 (-0.001, 0.002)	-0.000 (-0.001, 0.002)
BMIz	113	0.001 (-0.001, 0.002)	0.568	103	0.001 (-0.001, 0.003)	0.001 (-0.001, 0.003)	0.001 (-0.001, 0.003)	0.001 (-0.001, 0.003)
SBP (mmHg)	111	**0.015 (0.004, 0.026)**	**0.008**	101	**0.013 (0.003, 0.025)**	**0.013 (0.002, 0.024)**	**0.013 (0.002, 0.024)**	**0.015 (0.004, 0.026)**
DBP (mmHg)	111	**0.012 (0.004, 0.020)**	**0.005**	101	**0.013 (0.004, 0.022)**	**0.013 (0.003, 0.022)**	**0.013 (0.003, 0.022)**	**0.013 (0.004, 0.022)**
Cholesterol (mmol/L)	71	-0.001 (-0.002, 0.001)	0.241	65	-0.001 (-0.002, 0.001)	-0.001 (-0.002, 0.001)	-0.001 (-0.002, 0.000)	-0.001 (-0.002, 0.001)
HDL (mmol/L)	71	0.000 (-0.000, 0.001)	0.238	65	0.000 (-0.000, 0.001)	0.000 (-0.000, 0.001)	0.000 (-0.000, 0.001)	0.000 (-0.000, 0.001)
LDL (mmol/L)	71	-0.001 (-0.002, 0.000)	0.065	65	**-0.001 (-0.002, -0.000)**	**-0.001 (-0.003, -0.000)**	-0.001 (-0.002, 0.000)	**-0.001 (-0.003, 0.000)**
Triglycerides (mmol/L)	71	-0.000 (-0.000, 0.000)	0.752	65	-0.000 (-0.000, 0.000)	-0.000 (-0.000, 0.000)	-0.000 (-0.001, 0.000)	-0.000 (-0.001, 0.000)
FPG (mmol/L)	71	**0.001 (0.000, 0.000)**	**0.001**	65	**0.001 (0.000, 0.001)**	**0.001 (0.000, 0.001)**	**0.001 (0.000, 0.001)**	**0.001 (0.000, 0.001)**
Insulin (uU/ml)	71	**0.003 (0.000, 0.006)**	**0.046**	65	0.002 (-0.001, 0.006)	0.002 (-0.001, 0.006)	0.002 (-0.001, 0.005)	0.002 (-0.001, 0.006)
Vitamin D (nmol/L)	71	-0.06 (-0.112, -0.003)	0.062	65	-0.060 (-0.131, 0.011)	-0.058 (-0.131, 0.014)	-0.050 (-0.118, 0.016)	-0.06 (-0.121, 0.009)

All the models (sitting time as predictor variable) were adjusted for age, sex, BMIz, season and accounted for clustering by school. Model A: Minimally adjusted model; Model B: adjusted for LIPA; Model C: adjusted for Low-LIPA; Model D: adjusted for High-LIPA and Model E: adjusted for MVPA. Values in the table are displayed in bold when the association *p*-value is < 0.005

*Abbreviations* BMIz = body mass index z-score; WCz = waist circumference z-score; SBP = Systolic Blood Pressure; DBP = Diastolic Blood Pressure; HDL = High-Density Lipoprotein; LDL = Low-Density Lipoprotein; FPG = fasting plasma glucose; Vitamin D = 25hydroxyvitaminD; LIPA = Light Intensity Physical Activity (Total); low-LIPA = Low Light-Intensity Physical Activity (101 − 3.99 METS); low-LIPA = Low Light-Intensity Physical Activity (101–799 counts per minute); high-LIPA = High Light-Intensity Physical Activity (800 counts per minute– 3.99 METS); MVPA = Moderate- to- Vigorous-intensity Physical Activity (≥ 4 METS)

### Associations of sedentary time with health risk biomarkers

There was a negative association with WCz and BMIz only when the models were adjusted by MVPA. Sedentary time was positively associated with triglycerides but attenuated after adjusting for MVPA (Table 3).

**Table 3:** Associations between sedentary time and cardiometabolic risk factors prior to and after adjustment for moderate to vigorous physical activity intensity

Dependent variable:		Model A	*p*		Model B: MVPA
	n	β (95% CI)	*value*	n	β (95% CI)
WCz	335	-0.000 (-0.002, 0.002)	0.727	335	-0.003 (-0.005, -0.000)
BMIz	334	-0.001 (-0.003, 0.002)	0.565	334	-0.003 (-0.006, -0.000)
SBP (mmHg)	323	-0.000 (-0.021, 0.020)	0.968	323	0.002 (-0.023, 0.027)
DBP (mmHg)	323	0.011 (-0.007, 0.031)	0.222	323	0.005 (-0.017, 0.029)
Cholesterol (mmol/L)	159	0.001 (-0.001, 0.003)	0.334	159	0.002 (-0.002, 0.004)
HDL (mmol/L)	159	-0.000 (-0.001, 0.001)	0.522	159	0.000 (-0.000, 0.001)
LDL (mmol/L)	159	0.001 (-0.001, 0.003)	0.405	159	0.000 (-0.002, 0.003)
Triglycerides (mmol/L)	159	**0.001 (0.000, 0.001)**	**0.018**	159	0.001 (-0.000, 0.001)
FPG (mmol/L)	159	-0.000 (-0.002, 0.002)	0.677	159	-0.001 (-0.002, 0.002)
Insulin (uU/ml)	159	0.005 (-0.008, 0.020)	0.449	159	0.003 (-0.011, 0.019)
Vitamin D (nmol/L)	159	-0.089 (-0.191, 0.011)	0.08	159	-0.055 (-0.147, -0.037)

All the models (sedentary time as predictor variable) were adjusted for age, sex, season and BMIz and accounted for clustering by school. Model A: Minimally adjusted model; Model B: adjusted for MVPA. Values in the table are displayed in bold when the association *p*-value is >0.005

*Abbreviations* BMIz = body mass index z-score; WCz = waist circumference z-score; SBP = Systolic Blood Pressure; DBP = Diastolic Blood Pressure; HDL = High-Density Lipoprotein; LDL = Low-Density Lipoprotein; FPG = fasting plasma glucose; Vitamin D = 25hydroxyvitaminD; MVPA = Moderate- to- Vigorous-intensity Physical Activity (≥ 4 METS)

### Moderation analysis

No significant interactions between sitting (i.e. SBP, *p* = 0.446; DBP, *p* = 0.958; LDL, *p* = 0.768; FPG, *p* = 0.160; and insulin, *p* = 0.117) and sedentary time (i.e. WCz, *p* = 0.569; BMIz, *p* = 0.599; and triglycerides, *p* = 0.388) and any of the physical activity intensity variables were found for the significant models (prior and after the adjustment for physical activity intensity).

## Discussion

This cross-sectional study aimed to examine associations between *activ*PAL-measured sitting time and cardiometabolic risk factors and to determine whether ActiGraph-measured LIPA and MVPA moderated the relationship between sitting and cardiometabolic risk factors among children. Sitting time was unfavourably associated with SBP, DBP, and FPG and these associations persisted after adjusting for each PA intensity separately. There was a trend for sitting time to be detrimentally associated with insulin, however, this association was attenuated after adjusting for the physical activity intensity variables. Also, there was a negative association with LDL, in the unexpected direction, in the LIPA (i.e. total LIPA and low-LIPA) and MVPA adjusted models. When sedentary time was the predictor, an unfavourably association was observed between WCz and BMIz, but only in models adjusted for MVPA. Sedentary time was positively associated with triglycerides; however, this association weakened after adjusting for MVPA. There was, however, no significant moderation effects of LIPA (total, low-LIPA, high-LIPA) nor MVPA on associations between children’s sitting and sedentary time and cardiometabolic risk factors, which was inconsistent with previous research in adolescents.

While the sample size was small, to our knowledge, this is the first study to show a significant, detrimental association between device-based measures of sitting time and BP and FPG, among children aged 7–10 years. A previous study that measured sitting time using *activ*PALs in older children (11–12 years) found no association with cardiometabolic risk outcomes after adjusting for MVPA [20]. Although demographically comparable, the total volume of MVPA by Stockwell (2019) was double that recorded in the current study (90 vs. 38.2 min/day). The amount of time spent on MVPA in the current study is consistent with the amount of MVPA that has previously been reported for this age group [39]. The total amount of MVPA that might be needed to attenuate the association of sitting on FPG (e.g. 60 min for adults [40]) is arguably unrealistic based on population prevalence data; therefore, it is necessary to further explore if engaging in more LIPA and/or MVPA could benefit health among young people. For example, studies using isotemporal substitution to explore the effects of replacing sedentary time with PA of different intensities have found that replacing sedentary time with MVPA, but not LIPA, was favourably associated with body fat percentage [41]. Longitudinal and experimental research with larger sample sizes and similar methods to measure sitting time (*activ*PAL) are needed to determine if the findings of this study are spurious or reflect the actual associations of sitting time and cardiometabolic health makers among children.

The current study found an unfavourable association between sitting time and SBP and DBP, which also contrasts previous research [20]. The mechanisms responsible for the potential impact of excessive sitting and BP are unclear (e.g. sitting-induced reductions in blood flow and an increase in the accumulation of extravascular fluid in the legs [42] and reductions in conduit vessel flow and elevations in vasoactive mediators [42]), and there is limited evidence of this relationship among children. Previous studies, indicate a positive association between sedentary time [20, 43], using hip-mounted devices, and higher levels of SBP/DBP in children and adolescents. However, contrasting findings exist, with some studies reporting null associations [44–46], highlighting the complex relationship between sitting/sedentary time and blood pressure in youth. Longer-term BP monitoring and experimental research are needed to explore the association between sitting time and BP. A weak unfavourable association between sitting time and insulin have been found in this study and similar to the finding among adults [47], however this association has not been found among adolescents [48]. The unexpected association with LDL observed in our study warrants careful consideration. While the explanation remains unclear, several factors could contribute to this unexpected finding. For example, the relationship between sitting time and LDL may be influenced by a complex interplay of factors, such as dietary habits, genetic predispositions, and overall lifestyle. In addition, the inclusion of LIPA in the model may have affected the results due to sitting time and LIPA often being highly inversely correlated. It may be that children who engage in higher levels of LIPA accumulate different patterns of sitting, which are known to have varied relationships with health outcomes. Further exploration of these factors may provide further insights.

This study found no associations between sitting and the remaining cardiometabolic risk factors (i.e. WCz, BMIz, Cholesterol, HDL, Triglycerides and serum 25[OH]D), which is consistent with previous literature [12]. Only one previous study showed that prolonged sitting (bouts ≥ 30 min) was negatively associated with adiposity markers and positively associated with HDL cholesterol [20]. However, participants in the study by Stockwell et al. were several years older (aged 11–12 years) than those participants in our study. Therefore, the younger children in the present study may have had less exposure to higher volumes of sitting to impact cardiometabolic risk factors, although studies have shown an impact at early ages [29]. Importantly, children in the current study were generally healthy (normal BP, blood biomarkers and only *n* = 28 overweight/obese), therefore, finding associations between total volume sitting and health can be more challenging, and sitting patterns might play an important role in these associations. The adverse relationship between total volume of sedentary time and WC and BMI aligns, which was non-significant when sitting time was the predictor, with findings from prior cross-sectional and longitudinal studies [49]. However, while evidence suggests a connection between sedentary time and adiposity markers, it remains inconclusive and lacks evidence of causation [7]. Nonetheless, the evidence supporting the relationship between sedentary time and adiposity markers appears to be increasing over time [49]. The adverse association with triglycerides aligns with previous research [43]. For instance, Strizich et al. [44] demonstrated an association between sedentary time and triglycerides, which aligns with the findings of this study. However, unlike our study, they did not observe a similar relationship with HDL.

In the current study, children spent 59% of their day sitting, which is consistent with previous studies that have reported between 57 and 78% of waking time spent sitting, measured with *activ*PALs [31, 50, 51]. Children in this study also spent 40% of their waking hours in LIPA and only 4% in MVPA; however, neither LIPA nor MVPA moderated the relationship between sitting and cardiometabolic risk factors. In a previous study, sitting time was not associated with adiposity markers (BMIz and WCz), but LIPA and low-LIPA showed to moderate the detrimental association between sitting and BMIz and WCz in adolescents [21]. The inconsistent findings between this study in children and adolescents could be partly because adolescents had a higher volume of sitting (i.e., 59% vs. 68% of the day, respectively) and less volume of LIPA (27% vs. 39%, respectively). It is also possible that the very low percentage of waking hours spent in MVPA (∼4%) is not enough to overcome the effects of two-thirds of the day spent sitting by the children. Experimental research is needed to understand if, for example, replacing sitting time with LIPA is beneficial for children and adolescents in the long term.

This study found no associations between sitting and sedentary time and serum 25[OH]D when the models were adjusted for the season in which the assessment was conducted. To our knowledge, no previous studies have examined the relationship between device-assessed sitting time and circulating vitamin D in children. However, a previous study suggested that higher self-reported screen time was related to reduced serum concentrations of 25[OH]D in 8–9 year olds (*n* = 1464, not adjusted for MVPA) [52], but not 12–17 year olds (*n* = 1152, adjusted for MVPA) [53]. It could be that the deleterious impacts of sitting, which might be attributed to lower sun exposure due to sitting indoors, is counterbalanced with higher intensity physical activities (e.g. outdoor play/MVPA), which is likely to be performed outdoors (Cleland et al., 2010). Furthermore, considering that 72% of the data was collected during summer and autumn, significant sun exposure likely occurred during this study. Participation in outdoor play and MVPA is typically greater amongst children than adolescents, which may also partly explain the findings in the current study. Further research focusing on associations between sitting and vitamin D is needed to clarify the role of PA (i.e. indoors v outdoors). In contrast to previous cross sectional and longitudinal research (e.g. increase in sedentary time was unfavourably associated with changes in cardiometabolic score, triglycerides and DBP) [6], this study did not find any associations with other cardiometabolic makers (i.e. BP, Cholesterol, HDL, LDL, FPG, serum 25[OH]D and insulin). This divergence underscores the complexity of the relationship between sedentary behaviour and cardiometabolic health, potentially influenced by various factors such as sample characteristics, measurement techniques, and confounding variables.

Strengths of this study included the use of device-based assessments of sitting time (i.e. postural detection device) and PA of different intensities (i.e. actigraphy), the inclusion of a range of cardiometabolic risk factors among children, as well as the use of WC as a measure of central adiposity, given that BMI, while widely used, does not distinguish lean and fat mass. The limitations of this study include the use of cross-sectional data collected in 2010 and the small sample size, which may limit statistical power and the generalisability of the findings to the wider population. Nonetheless, this study contributes uniquely to the literature by employing both *activ*PAL and ActiGraph devices in children to explore their associations with health markers and the moderation effects of physical activity. However, while the data were collected in 2010 and patterns of sitting (and activities undertaken while sitting) may have changed, the device used to capture sitting time has undergone minor changes and relationships between volumes of sitting time and health outcomes are likely to be constant [54]. The study included a generally healthy sample, which may limit the ability to find associations with health outcomes. A further limitation is that participants had to remove the monitors for water-based activities. It is possible that physical activity levels were underestimated as water activities were not included. Use of waterproof wearable monitors may overcome this limitation in future studies. Given the study’s limitations and the scarcity of literature with which to compare our findings, the results should be considered cautiously.

## Conclusions

Our findings indicate that excessive sitting time is associated with some negative associations on cardiometabolic risk factors in children, suggesting that reducing the amount of time spent sitting could be a potential intervention to improve cardiometabolic health outcomes in children. However, further longitudinal and experimental studies are required to elucidate the relationship between sitting time and cardiometabolic health outcomes in children, and to inform targeted interventions to mitigate the adverse effects of sitting and sedentary time on health outcomes in children. Also, to examine the interplay between sitting time and different intensities of physical activity in relation to cardiometabolic risk factors also warranted.

### Electronic supplementary material

Below is the link to the electronic supplementary material.

Supplementary Material 1

## Data Availability

The dataset used for the current study could be made available on reasonable request, as the datasets are not publicly available due to ethical restrictions (participants have not consented to the use of their data for purposes other than those of which they originally consented)

## Abbreviations

BMI: body mass index
BP: blood pressure
DART: Diabetes Australia Research Trust
DBP: diastolic blood pressure
FPG: fasting plasma glucose
HDL: high-density lipoprotein
High-LIPA: high light-intensity physical activity
LDL: low-density lipoprotein
LIPA: light-intensity physical activity
Low-LIPA: low light-intensity physical activity
METs: metabolic equivalents
MVPA: moderate- to vigorous-intensity physical activity
NHMRC: National Health and Medical Research Council
PA: physical activity
SBP: systolic blood pressure
SEIFA: Socio-economic Index for Areas
WC: waist circumference
25[OH]D: 25-hydroxyvitaminD
