# Supplementary material for: The association between device-measured sitting time and cardiometabolic health risk factors in children
Source: BMC Public Health. 2024 Apr 12;24:1015. doi: 10.1186/s12889-024-18495-w (PMC11010425; doi:10.1186/s12889-024-18495-w)
Supplement: Supplementary file 1 — Supplementary Material 1 [file 12889_2024_18495_MOESM1_ESM.docx]

**Supplementary Material 1: Biomarker ranges provided by Melbourne Pathology as acceptable**

| Test | Range | Units | Percentage of participants in the normal range (%) |
| --- | --- | --- | --- |
| S Cholesterol | 2.4-4.5 | mmol/L | 52.4 |
| S Trig | 0.4-1.5 | mmol/L | 95.28 |
| S HDL-Chol | >1.20 | mmol/L | 91.51 |
| S LDL-Chol | <3.5 | mmol/L | 91.9 |
| Glu(FAST) | 3.6-6.0 | mmol/L | 98.1 |
| S Insulin-Fa | 0-17 | uU/mL | 98.6 |
| S 25-Hydroxy VIT D | 75-250 | nmol/L | 41.5 |
